# Excessive pyroptosis mediates the exacerbation of pneumonia caused by low-lethality influenza virus and secondary MRSA co-infection

**DOI:** 10.1038/s41420-026-03031-z

**Published:** 2026-04-02

**Authors:** Zi-Chen Tian, Yang Liu, Yi-Jun Niu, Xin Ai, Su-Ya Lao, Wei-Ming Xu, Xiao-Tong Lin, Cheng-Jie Xia, Zhi-Xuan Cai, Hai-Yan Zhu, Xun-Long Shi

**Affiliations:** School of Pharmaceutical Sciences, Shanghai Engineering Research Center of Immunotherapeutics, Fudan University, Shanghai, China

**Keywords:** Infectious diseases, Immune cell death, Antimicrobial responses

## Abstract

Co-infection with influenza A virus (IAV) and methicillin-resistant *Staphylococcus aureus* (MRSA) often causes severe pneumonia clinically; however, the role of innate immunity in this setting remains poorly understood. In our study, we established a murine co-infection model using low-lethality IAV and MRSA. Compared with single IAV or MRSA infection, co-infection with relatively low-lethality IAV and MRSA resulted in more severe pneumonia. Transcriptomic analysis indicated marked upregulation of genes involved in the pyroptotic signaling pathway. Consistently, flow cytometry and immunofluorescence analyses revealed caspase-1 activation and colocalization of gasdermin D (GSDMD) with macrophages in the lung. The RAW264.7 macrophage cell line was used for in vitro validation. Co-infection significantly enhanced the cleavage of caspase-1 and GSDMD in RAW264.7 cells. Furthermore, disulfiram, a pyroptosis inhibitor, was incorporated into the antiviral and antibacterial combination treatment. Although combined oseltamivir and linezolid treatment failed to fully alleviate lung injury, the inclusion of disulfiram, a GSDMD pore formation inhibitor, significantly ameliorated pneumonia symptoms and reduced inflammatory responses. Collectively, our findings highlight that macrophage pyroptosis contributes to the exacerbation of pneumonia induced by IAV and secondary MRSA co-infection. Inhibition of GSDMD-mediated pyroptosis may represent a viable therapeutic approach to alleviate disease severity and improve outcomes in lethal co-infection.

## Introduction

Influenza viruses, members of the *Orthomyxoviridae* family, are negative-sense, single-stranded RNA viruses. Since the 1918 influenza pandemic, these viruses have undergone rapid diversification and have continued to pose a significant threat to human health worldwide over the past century [1]. It is estimated by the World Health Organization (WHO) that seasonal influenza is responsible for up to 650,000 respiratory-related deaths each year, wreaking havoc on health systems [2]. Meanwhile, bacteria and influenza virus co-infection has been recognized as a great threat to public health [3–9]. One study conducted by Yan-Ning Liu [3] and colleagues implied that, among 798 co-infection patients, nearly half of them (405/798) were viral–bacterial co-infections. Clinically, influenza A virus (IAV) and methicillin-resistant *Staphylococcus aureus* (MRSA) co-infection is one of the most common co-infection diseases [4, 8, 9]. Beyond its high incidence [8, 9], IAV-MRSA co-infection induces stronger pro-inflammatory responses, such as elevated levels of IL-6, compared with single infection, and frequently results in the exacerbation of pneumonia and higher morbidity and mortality [4–6].

Recently, the mechanisms underlying viral-bacterial co-infections have been investigated extensively. Epithelial cell damage has been identified as one of the critical contributors to the increased severity of pneumonia during co-infection. Disruption of epithelium integrity can be observed after the incursion of severe acute respiratory syndrome coronavirus 2 (SARS-CoV-2), respiratory syncytial virus (RSV) and influenza viruses. Such damage facilitates bacterial invasion and ultimately leads to greater susceptibility to secondary infection with MRSA or other bacterial pathogens [10, 11]. Delayed epithelial repair has also been reported in various murine models of superinfection with influenza and *S.aureus*. Impaired epithelial cells regeneration and inhibition of homeostatic signaling pathway have been detected in these models, manifesting the hindered tissue repair process [12, 13].

Apart from these physiological mechanisms, the immune response, especially the innate immune system, plays a crucial role in the early stages of co-infection. Macrophages are among the most important innate immune cells and various studies have illustrated that macrophages dysfunction can contribute to the exacerbation of pneumonia during viral or bacterial infections [14–19]. Neupane et al. found that alveolar macrophages can patrol between alveoli and move directly towards inhaled bacteria in a healthy state. However, during IAV infection, the ability of alveolar macrophages crawling will be impaired and thus, unable to capture inhaled bacteria, leaving free bacteria in the bronchoalveolar lavage fluid (BALF) [14]. Besides, studies have confirmed that following exposure to IAV or bacteria, alveolar macrophages adopt a pro-inflammatory phenotype characterized by enhanced cytokine production and activation of inflammatory signaling pathways [15–17]. Moreover, accumulating evidence has revealed that IAV infection compromises the bacterial clearance capacity of alveolar macrophages, resulting in high susceptibility of secondary bacterial infection [18–20]. Although epithelial injury and impaired immune responses have been implicated in viral–bacterial co-infections, the contribution of inflammatory cell death, particularly macrophage pyroptosis, to IAV-MRSA co-infection pathogenesis remains unresolved.

Macrophages inflammatory cell death has also been proved to substantially contribute to lung injury and further aggravate macrophage dysfunction [21]. Pyroptosis is a highly inflammatory and immunogenic form of regulated cell death, which is dependent on caspase-1 activation. Activated caspase-1 can cleave gasdermin D (GSDMD), pro-interleukin-1β (pro-IL-1β), and pro-interleukin-18 (pro-IL-18) into their mature form. The cleaved N-terminal fragment of GSDMD forms membrane pores, and the release of IL-1β and IL-18 can amplify the inflammatory cascade and cause profound tissue injury [22]. Evidence suggested that macrophage pyroptosis occurs in Lipopolysaccharide (LPS)-challenged mouse models and induces lung inflammation and tissue damage [23, 24]. However, whether macrophage pyroptosis is induced during IAV and MRSA co-infection remains unclear.

In this study, we successfully established an IAV-MRSA co-infection mouse model. We discovered that treatment with oseltamivir and linezolid after the onset of clinical symptoms could not completely reverse tissue damage. Under these conditions, macrophage pyroptosis was evident, and the addition of disulfiram, a pyroptosis inhibitor, to oseltamivir and linezolid treatment could alleviate lung inflammation. Collectively, our findings suggest that targeting macrophage pyroptosis may represent a promising therapeutic strategy for IAV-MRSA co-infection.

## Results

### Low-lethality Influenza A virus and MRSA co-infection induces disproportionately severe pneumonia

**Fig. 1:**
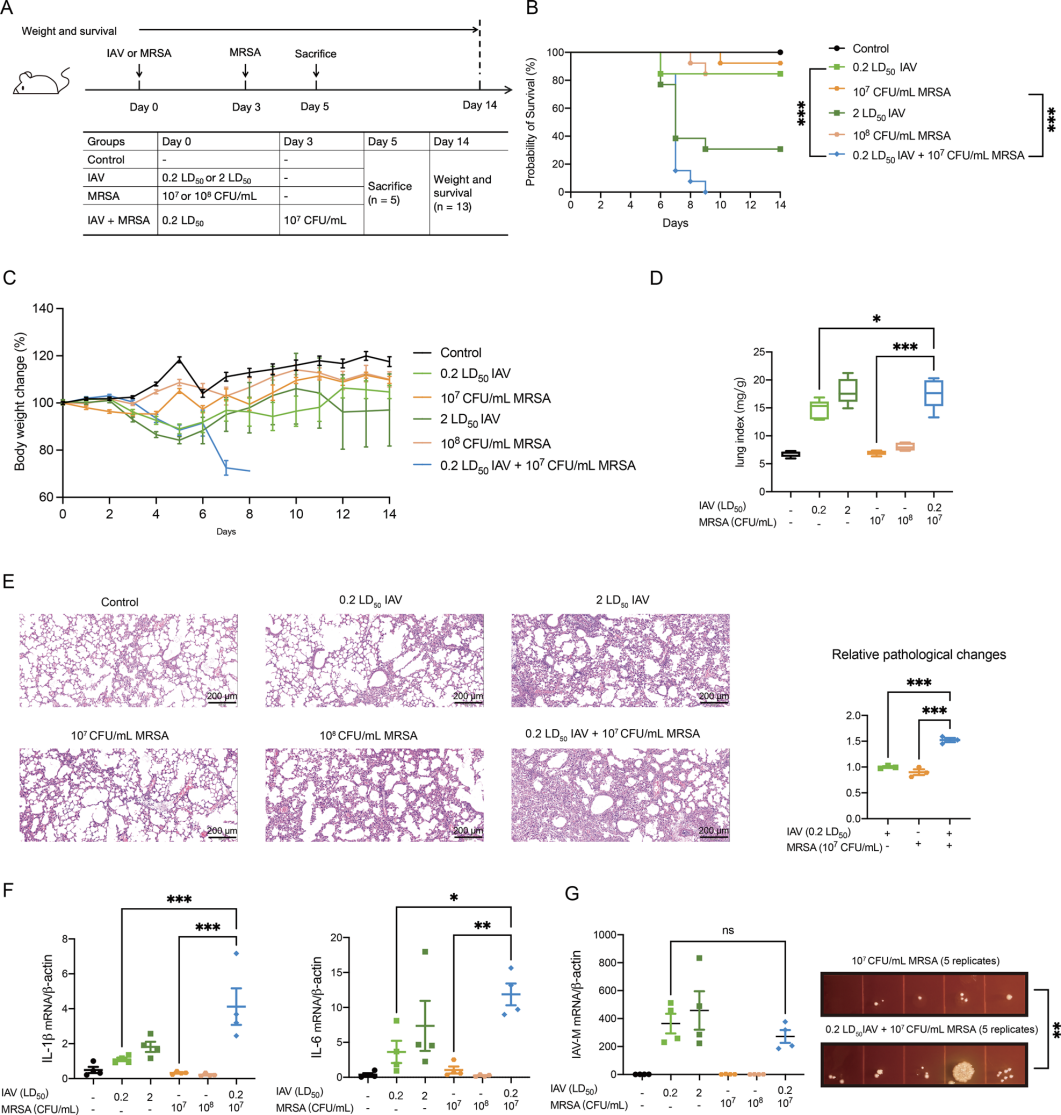
Pathological, inflammatory cytokines, viral load and bacterial burden analysis of IAV and MRSA co-infection mice. **A** Establishment of the single pathogen infection or co-infection model. Briefly, four-week-old C57BL/6 mice were infected with 0.2 LD_50_ IAV and then coinfected with 40 μL 10^7^ CFU/mL MRSA 3 days later. Control groups were only infected with single pathogen. **B** Survival curve of C57BL/6 mice infected with IAV and MRSA or separately (*n* = 13). **C** Weight changes of C57BL/6 mice infected with IAV and MRSA or separately (*n* = 13). Body weight changes are normalized to the average body weight in each group on the first day and multiply by 100%. **D** Lung index was calculated in the control and infectious groups (*n* = 5). Lung index = lung weight of mice (mg)/body weight of mice (g). **E** Representative pathological images (Scale bars: 200 μm) of lung tissue in different groups. Stain areas of images were calculated using image j and then normalized to the average area of the 0.2 LD_50_ IAV infection group (*n* = 3). **F** Inflammatory cytokines IL-1β and IL-6 mRNA levels were measured by qRT-PCR (*n* = 5). Data are normalized to β-actin and presented as fold of gene expression in the infected groups compared to the control. **G** Viral load of IAV was determined by analysis of influenza virus M gene copy (*n* = 5). Bacterial burden was determined by spreading 5 μL of the lung homogenates on the selective *S. aureus* medium (*n* = 5). Data are mean ± standard error of the mean (*p < 0.05; **p < 0.01; ***p < 0.001). IAV Influenza virus; MRSA methicillin-resistant *S. aureus*.

To evaluate the severity of lung injury induced by IAV and MRSA co-infection, C57BL/6 mice were infected intranasally (i.t.) with 0.2 LD_50_ IAV, and subsequently challenged three days later with 40 μl 10^7^ CFU/mL MRSA. For comparative analysis, parallel groups of mice received single infection with low or high doses of either pathogen (0.2 LD_50_ IAV, 2 LD_50_ IAV, 10^7^ CFU/mL MRSA or 10^8^ CFU/mL MRSA) (Fig. 1A). Survival rates and body weight changes were monitored for 14 days. Mice subjected to co-infection exhibited markedly reduced survival relative to all single-infection controls, including the high-dose IAV (2 LD_50_) and MRSA (10^8^ CFU/mL) groups (Fig. 1B). The most significant weight loss could also be observed in the co-infection group (Fig. 1C), indicating that IAV and MRSA co-infection can cause more critical disease manifestations, even more severe than a high dose IAV (2 LD_50_) or MRSA (10^8^ CFU/mL) monoinfection. Furthermore, co-infected mice also displayed significantly elevated lung index values (Fig. 1D), along with increased mRNA levels of pro-inflammatory cytokines IL-1β and IL-6 (Fig. 1F). Histopathological examination revealed preserved alveolar structures with mild infiltrates in single-infection groups, whereas co-infection induced substantial alveolar collapse accompanied by massive immune cell infiltration and hemorrhagic lesions (Fig. 1E). Interestingly, while viral RNA levels were not significantly higher in the co-infection group, pulmonary MRSA burdens increased markedly compared with MRSA alone (Fig. 1G), suggesting compromised antibacterial defense following IAV infection. Collectively, these evidence consolidate that IAV and MRSA co-infection precipitates disproportionately severe pneumonia characterized by exacerbated tissue damage and dysregulated inflammatory responses.

### Oseltamivir and linezolid combination therapy fails to sufficiently mitigate IAV-MRSA induced pneumonia

**Fig. 2:**
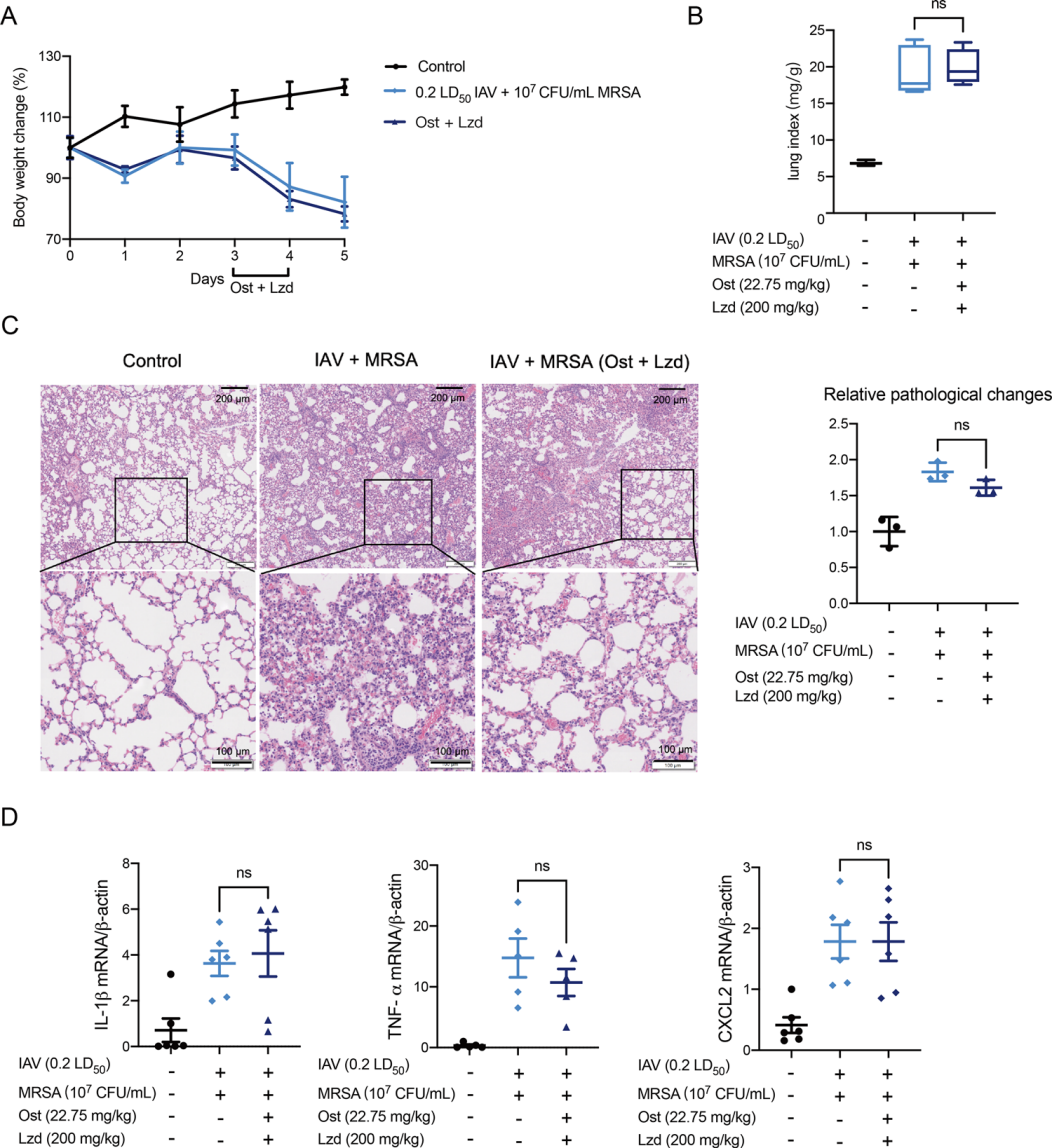
The effect of oseltamivir and linezolid combination treatment on co-infection mice. **A** Body weight changes of different groups were recorded for five days. Body weight changes are normalized to the average body weight in each group on the first day and multiply by 100% (*n* = 6). **B** Lung index was calculated in different groups (*n* = 6). Lung index = lung weight of mice (mg)/body weight of mice (g). **C** Representative pathological images (Scale bars: above 200 μm; below 100 μm) of lung tissue in different groups. Stain areas of images were calculated using image j and then normalized to the average area of the control group (*n* = 3). **D** Inflammatory cytokines IL-1β, TNF-α, and CXCL-2 mRNA levels were measured by qRT-PCR. Data are normalized to β-actin and presented as fold of gene expression in the infected groups compared to the control. Data are mean ± standard error of the mean (ns *p* > 0.05; *p < 0.05; **p < 0.01; ***p < 0.001). IAV Influenza virus, MRSA methicillin-resistant *S. aureus*, Ost oseltamivir, Lzd linezolid.

Linezolid, an orally active oxazolidinone antibiotic, is clinically effective against MRSA infections [25], while an in silico study showed that oseltamivir, a neuraminidase inhibitor, exhibited efficacy in an Influenza-*Streptococcus pneumoniae* co-infection model [26]. Based on this evidence, we hypothesized that combined oseltamivir and linezolid treatment could reduce lung pathology in pneumonia caused by IAV and MRSA. Briefly, mice were infected with IAV and MRSA, and on days 3 and 4, oseltamivir and linezolid were administered intragastrically. Body weight loss remained comparable between the co-infection and treatment groups (Fig. 2A). Similarly, lung index showed no significant improvement following combination therapy (Fig. 2B), suggesting that severe pulmonary injury persisted. Alveolar damage accompanied by hemorrhage (Fig. 2C) and enhanced mRNA levels of pro-inflammatory cytokines, including IL-1β, TNF-α and CXCL2 (Fig. 2D), also remained evident in treated mice. These data demonstrated that oseltamivir and linezolid failed to completely alleviate pneumonia symptoms. The limited efficacy of the combination therapy could be attributed to the delayed use of oseltamivir and the exacerbation of inflammatory response. These results underscore the importance of elucidating the mechanisms driving uncontrolled inflammation in order to improve therapeutic outcomes in co-infection settings.

### NOD-like receptor signaling and pyroptosis associated molecules were markedly activated during co-infection

**Fig. 3:**
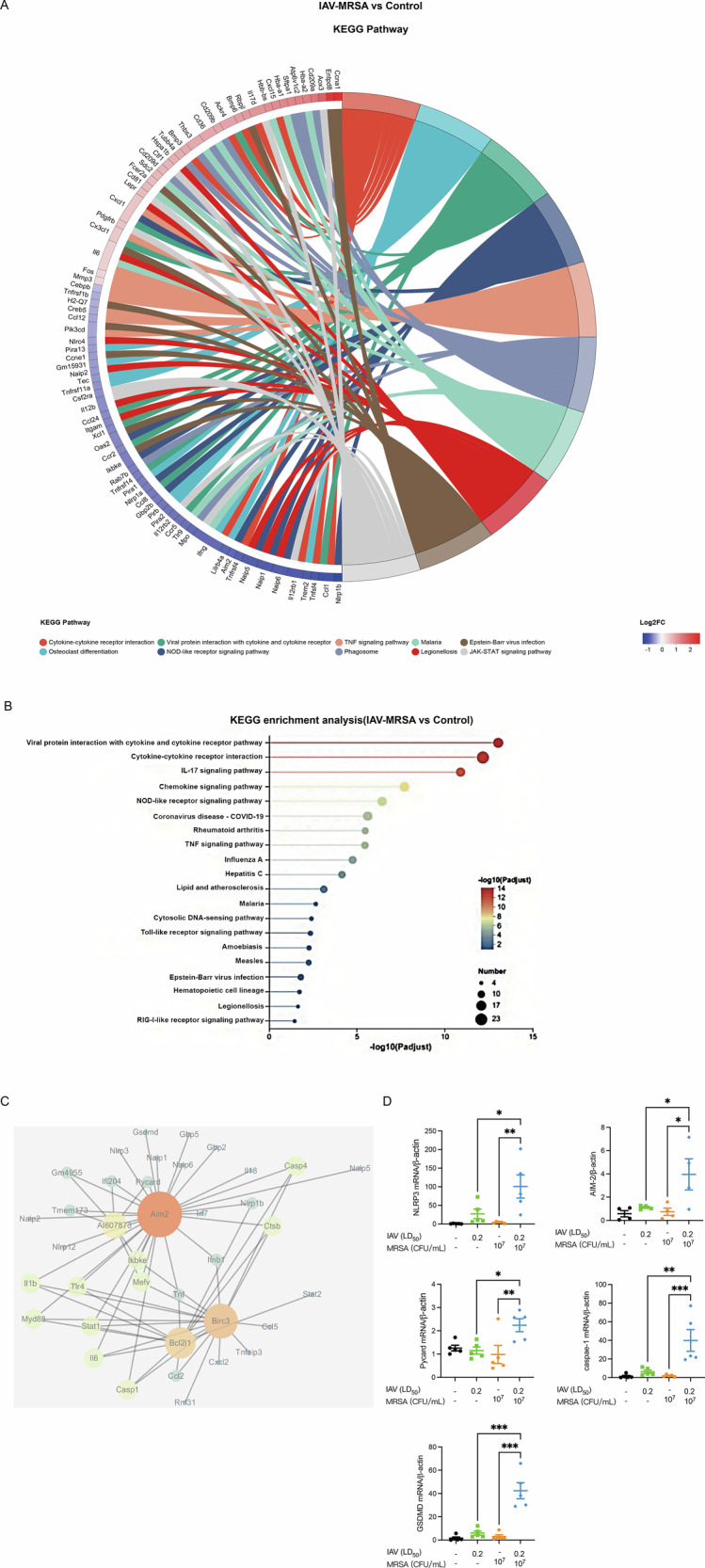
NOD-like receptor and pyroptosis signaling pathway were significantly upregulated during co-infection. **A** KEGG chord diagram and **B** KEGG enrichment analysis between the samples of the co-infection group and control group. **C** Hub proteins obtained from the PPI network. **D** NLRP3, AIM-2, Pycard, caspase-1, GSDMD mRNA levels were measured by qRT-PCR (*n* = 4 or 5). Data are normalized to β-actin and presented as fold of gene expression in different groups compared to the control group.

Given that combined antibiotic and antiviral therapy failed to control pneumonia severity, we next examined whether dysregulated host immune responses contribute to disease exacerbation. To investigate this, we conducted transcriptomic profiling on lung tissues from co-infected and control mice. KEGG chord diagram (Fig. 3A) and enrichment analysis (Fig. 3B) revealed substantial activation of inflammation-related pathways in the co-infection group. In addition to cytokine and chemokine signaling pathways, the NOD-like receptor signaling pathway was significantly upregulated. KEGG chord diagram also demonstrated that compared to the co-infection group, molecules associated with the NOD-like receptor signaling pathway were low expressed in the control group, suggesting that suppressing these inflammatory mediators may be an effective strategy to alleviate pneumonia. In the protein-protein interaction network analysis (Fig. 3C), core proteins, such as AIM2, Caspase-1, IL-1β and GSDMD were identified, indicating enhanced inflammasome assembly and subsequent release of pro-inflammatory cytokines during co-infection. Given the strong association of these proteins with pyroptosis, we then performed qRT-PCR to measure the mRNA levels of key molecules involved in pyroptosis in the lung (Fig. 3D). mRNA levels of pattern recognition receptors (PRRs) including NLRP3 and AIM2 were elevated. CARD- and PYD-containing adapter protein ASC was also upregulated, following by the increased expression of caspase-1 and GSDMD. Collectively, these data suggest that pyroptosis may play a significant role in the pathogenesis of co-infection-induced pneumonia.

### Macrophage pyroptosis plays a critical role in the development of co-infection induced pneumonia

**Fig. 4:**
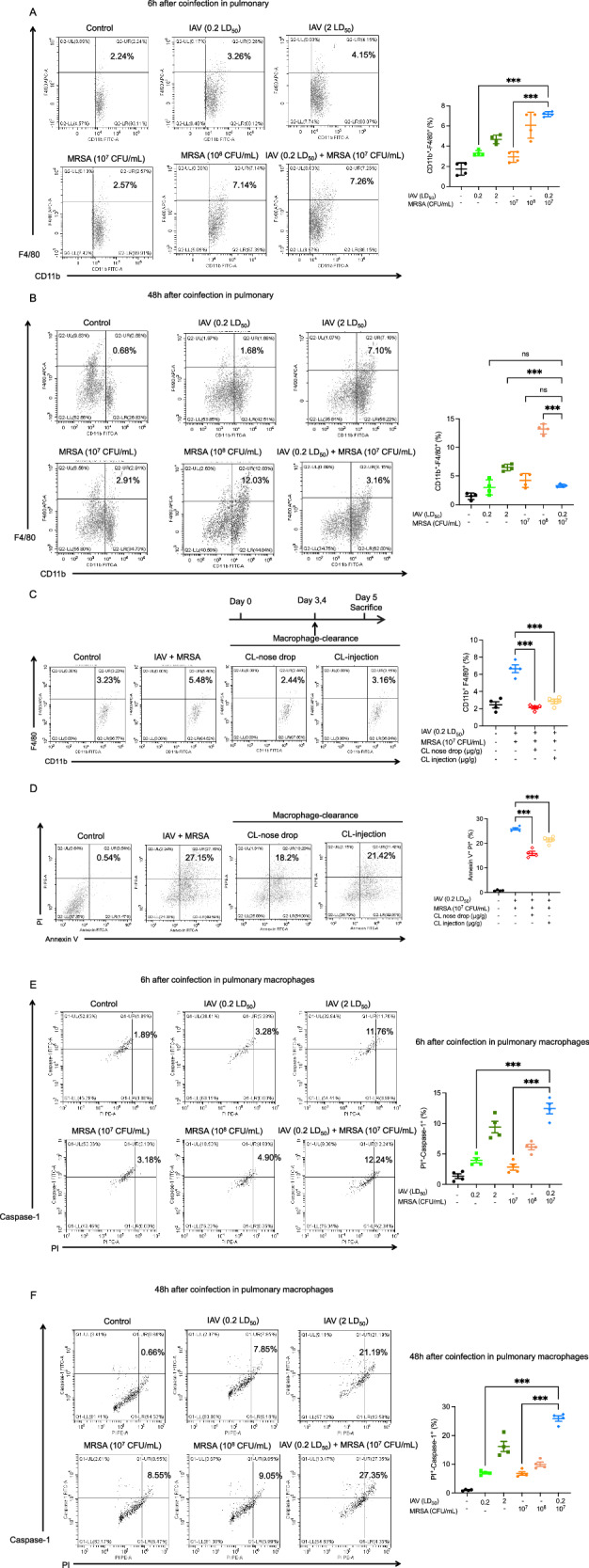
Macrophage pyroptosis was induced by co-infection and clearance of macrophage could reduce cell late apoptosis and necroptosis. **A** Flow cytometry analysis of the proportions of F4/80^+^ CD11b^+^ recruitment macrophage in pulmonary after 6 h co-infection and **B** after 48 h co-infection. **C** Macrophages were deleted by clodronate liposomes which were administrated intranasally or intraperitoneally. **D** Flow cytometry analysis of Annexin V^+^ PI^+^ ‌late apoptosis and ‌necroptosis cells. (*n* = 4) **E** Flow cytometry analysis of caspase-1^+^ PI^+^ pyroptosis macrophage in different groups after 6 h co-infection and **F** after 48 h co-infection. Data are mean ± standard error of the mean (*p < 0.05; **p < 0.01; ***p < 0.001) (*n* = 4). IAV Influenza virus, MRSA methicillin-resistant S. aureus, CL clodronate liposomes.

Macrophages are a kind of innate immune cells which play a crucial role in maintaining immunity homeostasis [27]. Previous studies have demonstrated that alveolar macrophages can shield bacteria from the immune system to avoid the induction of detrimental inflammation. However, their chemotactic ability can be compromised by the invasion of IAV and fail to control the progression of lung injury caused by secondary bacterial infection [14]. Thus, we hypothesized that macrophage dysfunction contributes to the exacerbated inflammation observed during IAV/MRSA co-infection. To assess macrophage dynamics in the lung, we first analyzed the recruitment of F4/80⁺CD11b⁺ macrophages. Six hours after co-infection, macrophage infiltration was significantly elevated compared to single infection groups (Fig. 4A). However, by 48 h post-infection, no significant difference was observed among the groups (Fig. 4B), indicating that macrophage death might have occurred. To investigate the functional relevance of macrophages in co-infection induced pneumonia, we depleted macrophages in vivo using clodronate liposomes. Efficient depletion was confirmed by flow cytometry (Fig. 4C), where the number of macrophages in the lungs of the co-infection groups markedly decreased after clodronate liposomes treatment. Our results demonstrated that macrophage depletion led to a reduction in the proportion of PI⁺ Annexin V⁺ cells in the lung (Fig. 4D), illustrating decreased late apoptosis and ‌necroptosis. These findings suggested that macrophages might contribute to tissue damage during co-infection. Meanwhile, flow cytometric analysis revealed a marked increase in caspase-1⁺ PI⁺ macrophages at both 6 h and 48 h post-infection (Fig. 4E, F), indicating the occurrence and progression of pyroptotic cell death in macrophages.

**Fig. 5:**
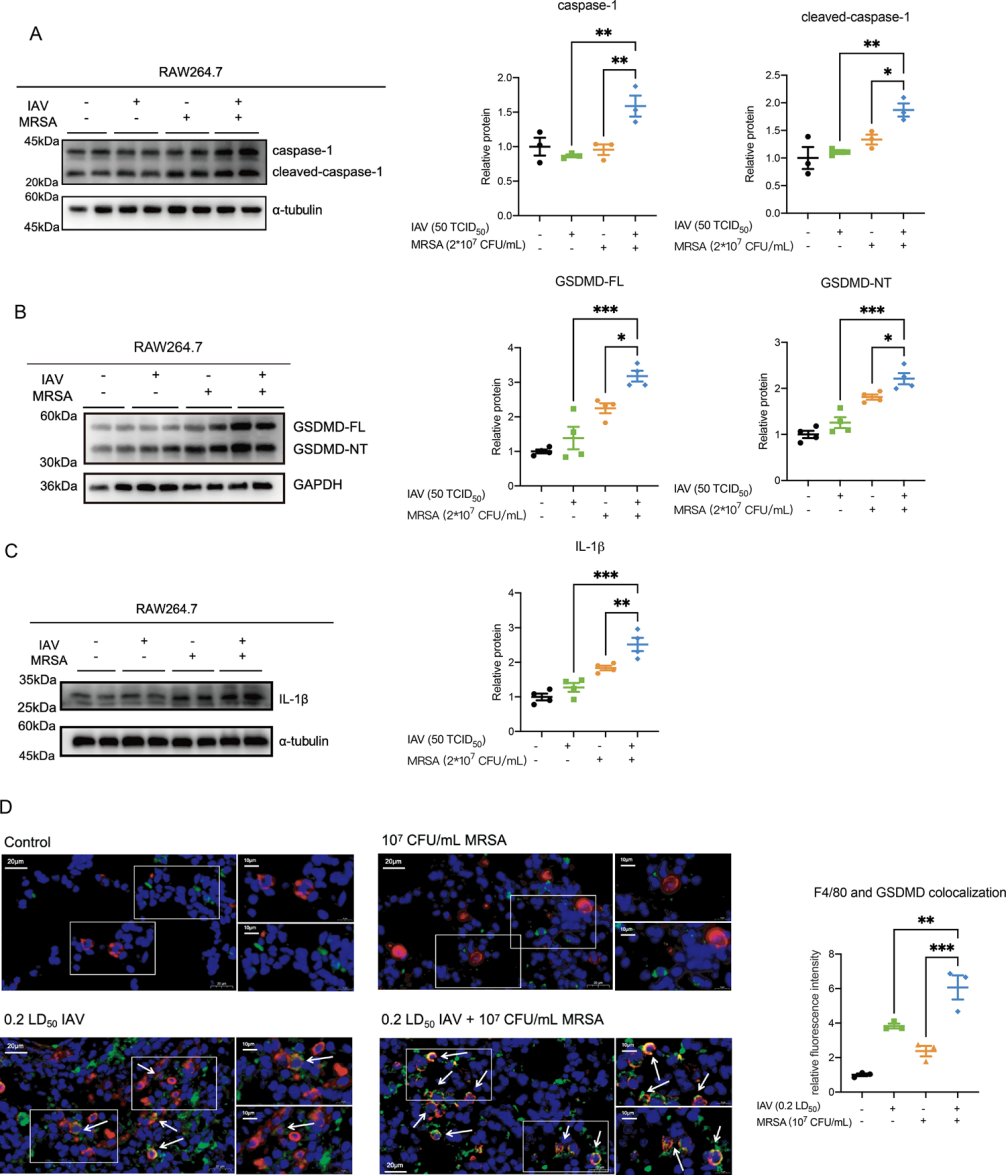
Macrophage pyroptosis was induced by IAV and MRSA co-infection in vitro and in vivo. **A** RAW264.7 was infected with IAV and MRSA. Cell lysates were immunoblotted with anti-caspase-1 and anti-α-tubulin antibodies. Relative protein levels were calculated. (*n* = 3) **B** Cell lysates were immunoblotted with anti-GSDMD and anti-GAPDH antibodies. (*n* = 4) **C** Cell lysates were immunoblotted with anti-α-tubulin and anti-IL-1β antibodies. (*n* = 4) **D** Immunofluorescence staining of F4/80 (red) and GSDMD (green), the scale bars are 20 μm(left) or 10 μm(right). Data are mean ± standard error of the mean (*p < 0.05; **p < 0.01; ***p < 0.001) (*n* = 3). IAV Influenza virus, MRSA methicillin-resistant *S. aureus*, GSDMD-FL Gasdermin D- full length, GSDMD-NT Gasdermin D-N terminal.

To validate these observations in vitro, we employed RAW264.7 macrophages to mimic co-infection. Western blot demonstrated increased levels of caspase-1 and its cleaved active form following IAV and MRSA infection (Fig. 5A). Furthermore, upregulation of GSDMD and its active form, Gasdermin D-N terminal (GSDMD-NT), was also detectable in RAW264.7cells (Fig. 5B). The expression of IL-1β was also elevated in the co-infection groups (Fig. 5C). Immunofluorescence staining of lung tissue revealed a more pronounced co-localization of macrophages (Red, F4/80) and GSDMD (Green) in the co-infection group, corroborating macrophage pyroptosis in vivo (Fig. 5D).

### Intranasal disulfiram administration mitigates macrophage pyroptosis and excessive inflammation

**Fig. 6:**
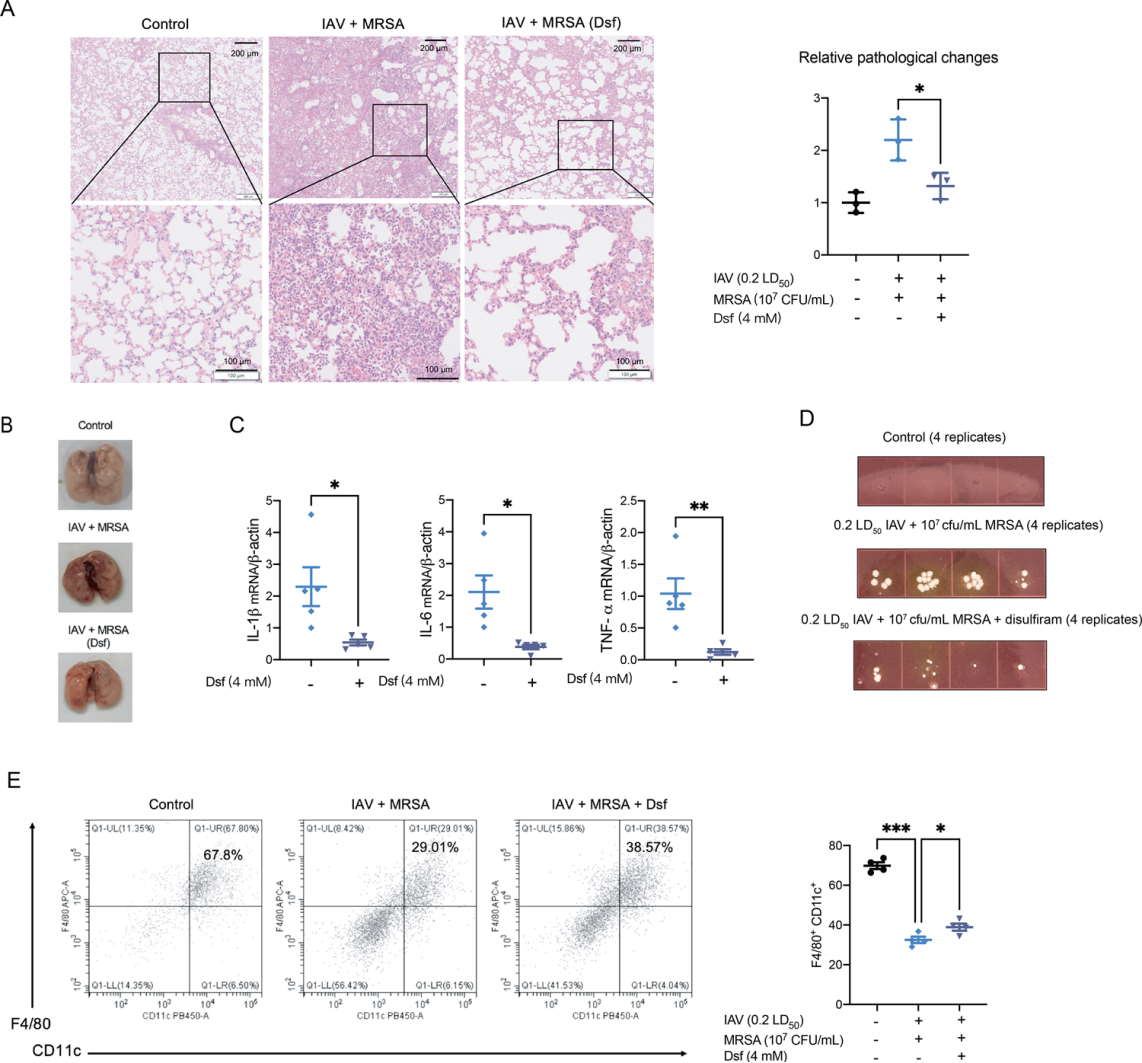
Disulfiram can partly attenuate tissue damage and harmful inflammatory response in IAV and MRSA co-infection. **A** Representative pathological images (Scale bars: above 200 μm; below 100 μm) of lung tissue in different groups. Stain areas of images were calculated using image j (*n* = 3). **B** Gross pathological changes of lung tissues were evaluated. **C** Inflammatory cytokines IL-1β, IL-6 and TNF-α mRNA levels were measured by qRT-PCR (*n* = 5). Data are normalized to β-actin and presented as fold of gene expression in the Dsf groups compared to the coinfected groups. **D** Bacterial burden was determined by spreading 5 μL of the lung homogenates on the selective *S. aureus* medium (*n* = 4). **E** Flow cytometry analysis of F4/80^+^ CD11c^+^ tissue-resident macrophages. Data are mean ± standard error of the mean (*p < 0.05; **p < 0.01; ***p < 0.001). IAV, Influenza virus; MRSA, methicillin-resistant *S. aureus*; Dsf, disulfiram.

**Fig. 7:**
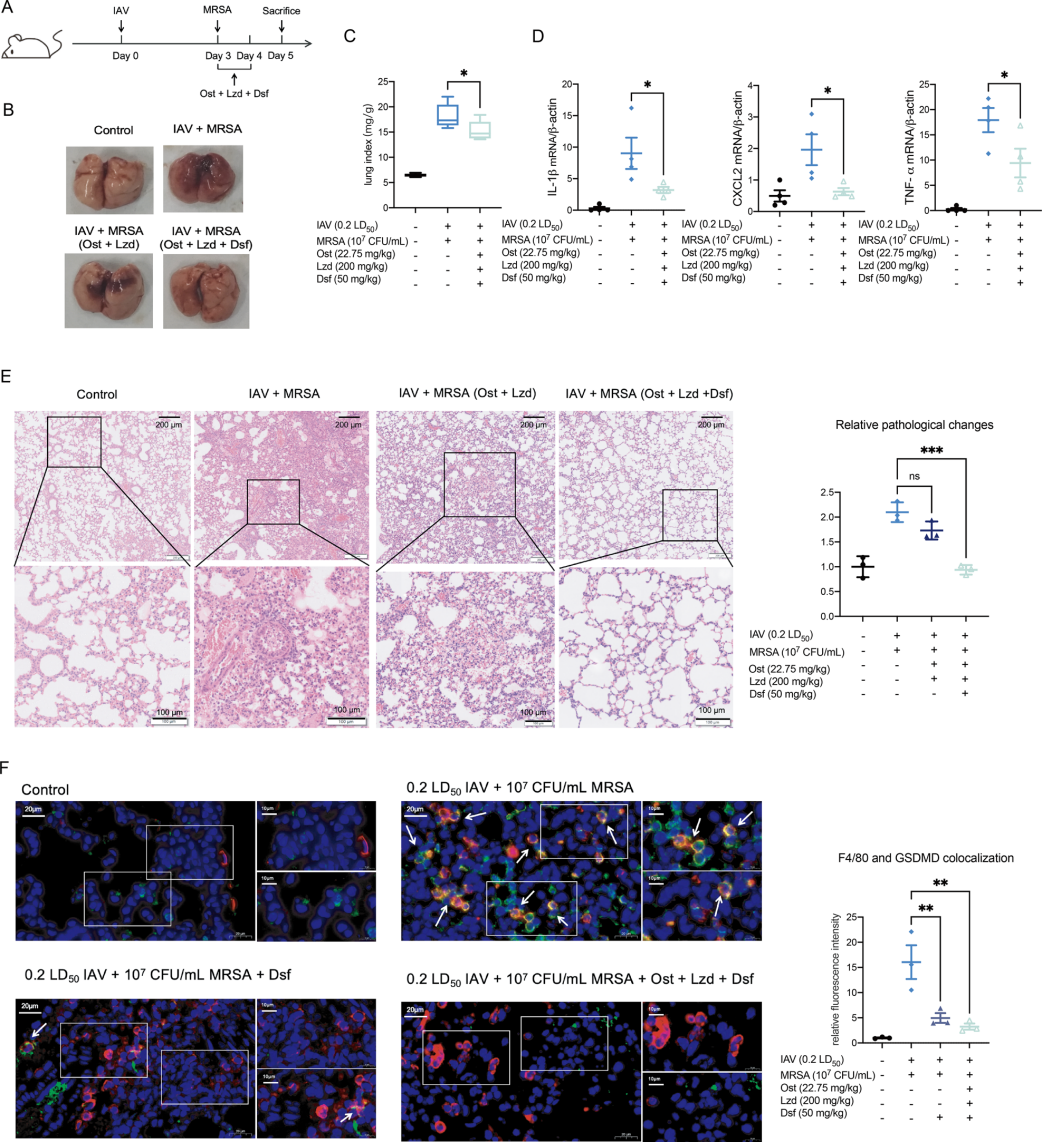
Combination treatment of oseltamivir, linezolid and disulfiram can inhibit macrophage pyroptosis in the lung and alleviate pneumonia. **A** Experimental scheme for assessing the combination treatment of oseltamivir, linezolid and disulfiram. **B** Gross pathological changes of lung tissues were evaluated. **C** Lung index was calculated in different groups (*n* = 6). Lung index = lung weight of mice (mg)/body weight of mice (g). **D** mRNA levels of IL-1β, CXCL-2 and TNF-α were measured by qRT-PCR (*n* = 4). Data are normalized to β-actin and presented as fold of gene expression in the infected groups compared to the control. **E** Representative pathological images (Scale bars: above 200 μm; below 100 μm) of lung tissue in different groups. Stain areas of images were calculated using image j and then normalized to the average area of the control group (*n* = 3). **F** Immunofluorescence staining of F4/80 (red) and GSDMD (green), the scale bars are 20 μm (left) or 10 μm (right). Data are mean ± standard error of the mean (*p < 0.05; **p < 0.01; ***p < 0.001). IAV Influenza virus, MRSA methicillin-resistant *S. aureus,* Ost oseltamivir, Lzd linezolid, Dsf disulfiram.

Disulfiram, a specific aldehyde dehydrogenase inhibitor, can be used to inhibit GSDMD pore formation in cell membrane, thus suppressing pyroptosis [28]. Multiple studies have confirmed its efficacy in attenuating pyroptosis under different circumstances [23, 29, 30]. To evaluate whether disulfiram could alleviate lung injury during IAV and MRSA co-infection, mice were firstly challenged with IAV followed by MRSA infection, and subsequently received intranasal disulfiram administration for three consecutive days. This treatment partially reduced tissue damage (Fig. 6A) and gross pathology revealed less congestion and pulmonary edema (Fig. 6B). Additionally, the mRNA levels of pro-inflammatory cytokines, including IL-1β, IL-6 and TNF-α, were significantly decreased following treatment (Fig. 6C). Meanwhile, the protein levels of GSDMD-NT in the lung tissue were downregulated in disulfiram treated groups (Supplemental Fig. 2). Bacterial burden was also decreased in the disulfiram treated group (Fig. 6D), suggesting a recovery of macrophage-mediated antibacterial capacity. To validate whether disulfiram attenuated pyroptosis of tissue-resident macrophages, we used flow cytometry to calculate the number of F4/80^+^ CD11c^+^ macrophages in the lung. F4/80^+^ CD11c^+^ macrophages decreased markedly in the co-infection groups compared to healthy controls, whereas disulfiram partially restored this macrophage population (Fig. 6E). Also, immunofluorescence staining of the lung showed reduced F4/80 and GSDMD co-localization following disulfiram treatment (Fig. 7F), indicating macrophage pyroptosis was inhibited by disulfiram. Collectively, these findings indicate that disulfiram exerts a protective effect against co-infection induced lung injury.

### Oseltamivir and linezolid combined with pyroptosis inhibitor disulfiram treatment can alleviate co-infection induced pneumonia more effectively

To determine whether targeting pyroptosis could enhance the therapeutic effect of antiviral and antibiotic treatment, these drugs were administered intragastrically for 2 days following IAV and MRSA co-infection (Fig. 7A). The combination therapy significantly reduced lung tissue damage, as evidenced by Fig. 7B, E, and resulted in a notable decrease in the lung index (Fig. 7C). The relative mRNA levels of pro-inflammatory cytokines, including IL-1β, CXCL-2 and TNF-α, were decreased in the combination-treated group (Fig. 7D). Immunofluorescence staining of lung tissue also indicated a significant reduction in the co-localization of macrophages and GSDMD after the combination treatment, manifesting the suppression of macrophage pyroptosis in the lung (Fig. 7F). In conclusion, while oseltamivir and linezolid cannot completely reverse the severe pneumonia caused by IAV and MRSA co-infection, the incorporation of disulfiram provides additional therapeutic benefit by mitigating excessive inflammation and pyroptotic injury.

## Discussion

Extensive evidence has confirmed that primary IAV infection followed by secondary bacterial invasion can cause severe pneumonia clinically [3, 4, 6, 10, 31]. Consistent with previous studies [5, 10], our findings supported that IAV and MRSA co-infection markedly amplified pulmonary inflammation (Fig. 1). However, the precise mechanisms driving such exacerbated pathology remain insufficiently understood. Previous studies focused on the disruption of epithelium adherence [10, 32] and alterations in mucus properties [11, 33], uncovering the significance of physiological mechanisms in the co-infection diseases. In addition, immunological studies have investigated how innate and adaptive immune responses are dysregulated during co-infection [14, 34, 35]. Despite this, the role of macrophage dysfunction, particularly pyroptotic cell death, has not been fully characterized in the context of IAV and MRSA co-infection. In our study, we concentrated efforts on macrophage pyroptosis in IAV and secondary MRSA infection. Macrophage pyroptosis was detectable both in vitro and in vivo (Figs. 3-5), contributing to excessive inflammatory cytokine production and aggravated lung injury. While oseltamivir and linezolid combination treatment failed to completely reverse the severe pneumonia caused by IAV and MRSA (Fig. 2), incorporating disulfiram, a GSDMD pore formation inhibitor, significantly alleviated tissue damage and improved survival outcomes (Figs. 6, 7).

Macrophages are highly plastic immune cells that undergo phenotype switching in response to pathogenic stimuli. Numerous studies have demonstrated that infection or environmental stress can drive macrophages toward a pro-inflammatory state. For instance, the exposure of cigarette smoke and IAV can induce a dual polarization phenotype in alveolar macrophages, contributing to the acute exacerbation of chronic obstructive pulmonary disease [36]. Following H1N1 infection, increased phosphorylated signal transducer and activator of transcription 1 (p-STAT1) was observable in RAW264.7 cells, which facilitated M1 macrophage polarization and increased expression of inflammatory genes [37]. Besides, stimulation with influenza virus and LPS, mimicking a co-infection model, could elevate macrophage inflammatory factor expression through the up-regulation of Nedd4L [15]. In addition, early exposure to *Mycobacterium tuberculosis* triggered higher expression of cell death related genes in M1 macrophages compared with M2 macrophages [38]. Collectively, these studies suggest that infectious conditions can drive macrophages toward a heightened pro-inflammatory phenotype. If this activated state is not properly controlled, it may precipitate excessive inflammation and contribute to worsening disease pathology.

Macrophage pyroptosis can exacerbate inflammatory reactions in many different pathological settings. Neutrophils have been shown to release exosomes containing specific microRNAs that prime macrophage pyroptosis, resulting in pronounced lung tissue injury [39]. Besides, gut microbiota changes, oxidized low-density lipoprotein and some circular RNAs could also lead to macrophage pyroptosis in various disease models [40–42]. However, in the context of IAV and MRSA co-infection, the upstream signals that initiate macrophage pyroptosis remain unknown and warrant further investigation. Notably, these pyroptotic macrophages can secret extracellular vesicles, promoting communications between different cells and amplifying inflammatory cascades. Consequently, these extracellular vesicles can activate the epithelial cells, induce vascular leakage, recruit neutrophils and accelerate formation of neutrophil extracellular traps [23, 43]. Also, whether extracellular vesicles derived from pyroptotic macrophages similarly mediate pneumonia exacerbation during IAV and MRSA co-infection remains an open question.

Preventing macrophage pyroptosis offers a promising strategy to control the excessive inflammatory responses associated with IAV and secondary MRSA infection. Natural compounds have demonstrated efficacy in limiting macrophage pyroptosis [44, 45]. Quercetin, a natural flavonoid, can inhibit the assembly of NLRP3 and AIM2 inflammasomes by disrupting the interaction between ASC and caspase-8, thereby suppressing macrophage pyroptosis [44]. Additionally, ubiquitin-independent degradation pathways can also downregulate macrophage pyroptosis. REGγ, a member of the proteasomal activators 11S family, could degrade a pro-apoptotic protein, Bim. Since Bim accumulation enhances gasdermin cleavage, its degradation can help block macrophage pyroptosis [46]. Beyond directly targeting pyroptosis-related proteins, modulation of extracellular vesicles derived from different cells can also inhibit macrophage pyroptosis [47, 48]. For instance, small interfering RNA targeting high-mobility group box 1 (siHMGB1) delivered via extracellular vesicles suppresses LPS-induced caspase-11 activation, and subsequently prevent macrophage pyroptosis [47]. Also, disulfiram, a specific aldehyde dehydrogenase inhibitor, has been employed to block GSDMD pore formation in the cell membrane [28]. In our study, we discovered that disulfiram could block macrophage pyroptosis in IAV and MRSA co-infection and alleviate pneumonia, indicating its potential as a therapeutic agent for infectious pneumonia. However, the limited water solubility of disulfiram poses challenges for its administration, particularly for targeted delivery to the lungs in pneumonia cases. Therefore, the development of advanced drug delivery systems, such as lipid nanoparticles (LNPs), is necessary to enhance pulmonary targeting efficiency and bioavailability of disulfiram, and thus warrants further investigation [49].

Overall, our data suggest that IAV and secondary MRSA infection causes severe pneumonia in mice, characterized by aggravated tissue injury, elevated bacterial burden, and excessive pro-inflammatory cytokine production. More importantly, our research, to the best of our knowledge, is the first time to identify macrophage pyroptosis as a key pathogenic mechanism driving lung damage during IAV and MRSA co-infection. We show that macrophage pyroptosis contribute to alveolar destruction, hemorrhage and excessive inflammatory responses in the lung, whereas inhibiting pyroptosis markedly alleviates disease severity. Therefore, these results prompt the potentiality of inhibiting pyroptosis to intervene the pathological process in IAV and MRSA co-infection and prevent the onset of lethal pneumonia. Nevertheless, the upstream pathways that trigger macrophage pyroptosis in the context of viral and bacterial co-infection remain poorly defined and warrant further mechanistic investigation.

## Methods and materials

### Animals and ethics statement

Four-week-old C57BL/6 female mice were purchased from Slaccas Company (Shanghai). All mice were bred and housed in a pathogen-free environment with freely available standard food and water at the Laboratory Animal Center of School of Pharmacy, Fudan University, China.

All animal experiments were approved and performed in compliance with the Animal Ethics Committee of the School of Pharmaceutical Sciences, Fudan University, China(2025-02-SY-SXL-13). All methods were performed in accordance with the relevant guidelines and regulations.

### Virus and bacteria

IAV H1N1 A/FM/1/47 (H1N1) was maintained at the center for anti-inflammation and anti-virus drug laboratory (School of Pharmacy, Fudan University, Shanghai, China). The influenza virus was serially diluted in RPMI 1640 medium (Meilunbio, Dalian, China) before use. The median lethal dose (LD_50_) was determined at a concentration of 5*10^-5^ dilution in the experiment mice. Methicillin-resistant *Staphylococcus aureus* (MRSA20-385) was acquired from Institute of antibiotics, Huashan Hospital Affiliated to Fudan University, China, and were maintained in laboratory of Department of Biological Medicines & Shanghai Engineering Research Center of ImmunoTherapeutics, School of Pharmacy, Fudan University.

### Establishment of animal infection

Newly purchased C57BL/6 mice were firstly kept in the pathogen-free environment for one day. Animals were randomly assigned to different experimental groups. Then, they were anesthetized with isoflurane using a gas anesthesia apparatus. For IAV infection, animals were infected with 0.2 LD_50_ or 2 LD_50_ IAV intranasally. For MRSA infection, animals were infected with 40 μL 10^7^ CFU/mL or 10^8^ CFU/mL MRSA intranasally. For co-infections, animals were firstly infected with 0.2 LD_50_ IAV, followed by 40 μL 10^7^ CFU/mL MRSA three days later. The weight change and the survival rate of mice within 14 days were recorded to evaluate the severity of IAV/MRSA co-infection.

To explore the therapeutic effects of Oseltamivir (Ost, Roche, Shanghai, China, J20140121) and Linezolid (Lzd, Aladdin, China, L126613) combination therapy on IAV/MRSA co-infection, mice were treated intragastrically with 22.75 mg/kg Ost and 200 mg/kg Lzd [50, 51] after the establishment of the co-infection. Also, to evaluate the effects of GSDMD pore formation inhibitor disulfiram (Dsf, MCE, China, Cat#HY-B0240) and these three drugs combination therapy on IAV/MRSA co-infection, mice were treated intranasally with 4 mM Dsf or intragastrically with 50 mg/kg Dsf combined with 22.75 mg/kg Ost and 200 mg/kg Lzd. Investigators responsible for outcome assessment were blinded to group assignments and data analysis was conducted using objective quantitative measures to minimize potential bias.

### Depletion of macrophage in vivo

Mice were infected with 0.2 LD_50_ IAV and after three days, were infected with 40 μL 10^7^ CFU/mL MRSA. Then 10 μg/g clodronate liposomes (Yeasen, 40337ES08) were injected intranasally or intraperitoneally twice on the next two days to clear macrophage in vivo.

### Hematoxylin–eosin (H&E) staining

The upper lobe of the mouse right lung was fixed in 4% paraformaldehyde for more than 24 h. Then the tissue was embedded in paraffin blocks and subsequently sectioned at 4 μm. After dewaxing and hydration, the sections were stained with hematoxylin and eosin. Histological images were acquired using an Olympus SLIDEVIEW VS200 microscope.

### Quantitative real-time PCR analysis (RT-qPCR)

Approximately 20 mg of the mouse lung tissue was placed in 200 μL Trizol (15596018, Invitrogen) and then homogenized at 50 Hz, 30 s for three times. Total RNA was extracted and reversely transcribed into cDNA using the cDNA synthesis mix (Beyotime, China, Cat#D7182L) according to the manufacturer instructions. Levels of mRNA encoding for IL-1β, IL-6, CXCL-2, TNF-α, IAV-M, AIM-2, NLRP3, Pycard, caspase-1, GSDMD were measured by real-time PCR using Universal SYBR Green Fast qPCR Mix (ABclonal, China, Cat# RK21203) in step one plus real time PCR system (Applied Biosystems, USA). The ratio for the mRNA was normalized to β-actin. The PCR amplification procedure included pre-denaturation at 95 °C for 3 min; each cycle included denaturation at 95 °C for 5 s, annealing at 60 °C and extension for 1 min, with a total of 40 cycles. The primer sequences are as following:

**Table:** 

Name	Sequence (5’ to 3’)
β-actin forward primer	CATTGCTGACAGGATGCAGAAGG
β-actin reverse primer	TGCTGGAAGGTGGACAGTGAGG
IL-1β forward primer	TGCCACCTTTTGACAGTGATG
IL-1β reverse primer	TGATGTGCTGCTGCGAGATT
IL-6 forward primer	CCAGAGATACAAAGAAATGATGG
IL-6 reverse primer	ACTCCAGAAGACCAGAGGAAAT
CXCL-2 forward primer	CAAGGGTTGACTTCAAGAACATCC
CXCL-2 reverse primer	CCTTGAGAGTGGCTATGACTTC
TNF-α forward primer	GCCTCTTCTCATTCCTGCTT
TNF-α reverse primer	TGGGAACTTCTCATCCCTTTG
IAV-M forward primer	GACCGATCCTGTCACCTCTGAC
IAV-M reverse primer	AGGGCATTCTGGACAAAGCGTCTA
AIM-2 forward primer	GTCACCAGTTCCTCAGTTGTG
AIM-2 reverse primer	CACCTCCATTGTCCCTGTTTTAT
NLRP3 forward primer	CGAGACCTCTGGGAAAAAGCT
NLRP3 reverse primer	GCATACCATAGAGGAATGTGATGTACA
Pycard forward primer	GCAACTGCGAGAAGGCTATG
Pycard reverse primer	GCTCCTGTAAGCCCATGTCT
caspase-1 forward primer	AGATGCCCACTGCTGATAGG
caspase-1 reverse primer	TTGGCACGATTCTCAGCATA
GSDMD forward primer	TTCCAGTGCCTCCATGAATGT
GSDMD reverse primer	GCTGTGGACCTCAGTGATCT

### Immunofluorescence

Paraffin-embedded 4 μm sections of lungs were used for immunofluorescence. After deparaffinization, sections were incubated in a citrate buffer solution for antigen retrieval. Then the slides were incubated with autofluorescence quencher solution (Wuhan Baiqiandu Biotechnology Co., Ltd, B0008) for 30 min and 3%H_2_O_2_ to inhibit endogenous peroxidase. Following 30 min blocking with 3% bovine serum albumin, slides were incubated at 4 °C for 24 h with primary antibody [GSDMD Rabbit pAb (ABclonal, China, Cat#A18281, 1:100); F4/80 (CST, USA, Cat#70076, 1:200)], which completely covered the tissue sample. The following day, sections were incubated with the secondary antibody for 1 h at room temperature. Finally, the samples were incubated with DAPI (Wuhan Baiqiandu Biotechnology Co., Ltd, B0011), autofluorescence quencher solution and sealed and examined under a confocal laser scanning microscopy.

### Flow cytometric analysis

Mouse lung tissue was broken manually and filtered through cell strainers (WoHong, Shanghai, WHB-40UM-S) to obtain single cell suspensions. The suspensions were centrifuged in 350 g for 5 min and treated with red blood cell lysis buffer (Beyotime, C3702). The single cell suspensions were blocked with BeyoFC^TM^ Fc Receptor Blocking Solution (Beyotime, C1755S) for 10 minutes on ice. Then the samples were stained with fluorophore-conjugated anti-mouse antibodies or dyes [CoraLite® Plus 405 Anti-Mouse CD11c (Proteintech, Cat No. CL405-65602); anti-mouse CD170 (Siglec-F) Antibody (BioLegend, Cat#155507); anti-mouse CD45 Antibody (ABclonal, Cat A27305); FAM-YVAD-FMK Caspase-1 inhibitor (Immunochemistry, Lot#22N19); anti-mouse F4/80 Antibody (ABclonal, A27478); anti-mouse CD11b Antibody (BioLegend, Cat#101205); PI (BioLegend, Cat#421301); Annexin V-FITC (Beyotime, C1062M)] and fixed with 4% paraformaldehyde. The suspensions were then washed with PBS. Finally, flow cytometry was performed on the Beckman CytoFlex S, and data was analyzed on CytExpert.

### Quantification of bacterial burden

Lung tissue was homogenized at 25 Hz, 3 min in PBS. And then 5 μL of the samples was spread onto MRSA selective medium (Hopebio, HB4128). Then the medium was incubated in 37 °C for 2 days, and the images of the medium were taken to determine the bacteria burden in the lung.

### RNA-Seq analysis

Sample preparation: Lungs from the control and IAV-MRSA groups were harvested for RNA-seq analysis at Shanghai Majorbio Biopharm Technology Co. Total RNA was extracted using TRIzol Reagent according the manufacturer’s instructions. Then RNA quality was determined by 5300 Bioanalyser (Agilent) and quantified using the ND-2000 (NanoDrop Technologies).

Library preparation: The lung RNA-seq transcriptome library was prepared following Illumina® Stranded mRNA Prep, Ligation (San Diego, CA) using 1 μg of total RNA. Shortly, messenger RNA was isolated by oligo(dT) beads and fragmented by fragmentation buffer. Double-stranded cDNA was synthesized with random hexamer primers. Libraries were size selected for cDNA target fragments of 300–400 bp use magnetic beads followed by PCR amplified for 10-15 PCR cycles. After quantified by Qubit 4.0, the sequencing library was performed on NovaSeq X Plus platform(PE150) using NovaSeq Reagent Kit.

Quality control and Read mapping: The raw paired end reads were trimmed and quality controlled by fastp with default parameters. Then clean reads were separately aligned to reference genome with orientation mode using HISAT2 software. The mapped reads of each sample were assembled by StringTie in a reference-based approach.

Differential expression analysis: After obtaining the gene read counts, differential gene expression analysis was performed across multiple samples (≥2) to identify differentially expressed genes (DEGs) between samples, followed by functional studies of these DEGs. The default criteria for identifying significantly differentially expressed genes were FDR < 0.05 and |log2FC | ≥ 1. A gene was considered a differentially expressed gene (DEG) only if it met both conditions simultaneously.

KEGG chord diagram and KEGG enrichment analysis described in this paper was performed on the free online platform Majorbio Cloud Platform (https://cloud.majorbio.com/). RNA-seq results of lungs in control and IAV-MRSA mice were from PRJNA1266226 datasets. Protein-protein interaction analysis was performed in https://string-db.org/.

### Cell culture and infection

The RAW264.7 cell line was obtained from the American Type Culture Collection (ATCC). RAW264.7 cells were cultured in the DMEM supplemented with 10% (v/v) heat-inactivated fetal bovine serum (FBS), 100 U/mL penicillin, and 100 mg/mL streptomycin (Gibco) at 37 °C under 5% CO2. Before infection, cells were seeded in six-wells plate with the concentration of approximately 4*10^5^ cells/mL. The next day cells were infected with 50 TCID_50_ of the IAV for 2 h (TCID_50_ was calculated using MDCK cells, and as RAW264.7 cells were not susceptible to IAV infection, a relatively high TCID_50_ was used to assist infection.), and continued to be cultured in the DMEM supplemented with 3% (v/v) heat-inactivated FBS for 22 h to assist the virus infection. Then the cells were infected with 2*10^7^ CFU/mL MRSA for 6 h and harvested for western blotting.

### Western blotting

Samples were lysed in RIPA buffer (Beyotime, P0013B) with protease inhibitors (Beyotime, P1045). Protein concentrations were determined using an enhanced BCA assay kit (P0009, Beyotime). Protein samples were separated by SDS gel electrophoresis according to different molecular mass, and then they were transferred onto PVDF membranes (Merck Millipore, ISEQ00010). The PVDF membranes carrying the protein samples were blocked for 15 min with QuickBlock^TM^ Blocking Buffer (Beyotime, P0252). Membranes were incubated at 4 °C overnight with Caspase-1Rabbit pAb (ABclonal, China, Cat#A0964, 1:1000); GSDMD Mouse mAb (Proteintech, China, Cat No. 66387-1-lg, 1:5000); IL-1β polyclonal antibody (Proteintech, China, Cat No. 26048-1-AP, 1:1000); α-tubulin Mouse mAb (ABclonal, China, Cat#AC012, 1:5000); GAPDH Mouse mAb (Proteintech, China, Cat#60004-1-lg, 1:50000). Then the membranes were washed with TBST solution and incubated for 1 h in secondary species-specific antibodies (Beyotime, A0216/A0208). Finally, membranes were detected in the gel imager (ProteinSimple, FluorChem) with an ECL luminescence regent kit (MeilunBio, MA0186).

### Statistical analyses

Data are shown as means ± Standard Error of the Mean. One-way analysis of variance (ANOVA) or student’s t-test, was performed using GraphPad Prism 9. Statistically significant differences are indicated as *P < 0.05, **P < 0.01 or ***P < 0.001. Investigators responsible for outcome assessment were blinded to group assignments and data analysis was conducted using objective quantitative measures to minimize potential bias. Inclusion and exclusion criteria were established prior to the experiments. Post hoc power analysis was conducted using the observed effect size and sample size. All the achieved statistical power was estimated to be above 80%, indicating that the study was adequately powered to detect the observed differences. All statistical tests were justified as appropriate and data met the assumptions of the tests. The number of animals and number of independent experiments used are indicated for each experiment in the figure legends.

## Supplementary information

Supplemental figure 1. Immunofluorescence image for Figure.5D and Figure.7F under 20× magnification.

Supplemental figure 2. Lung lysates were immunoblotted with anti-GSDMD and anti-α-tubulin antibodies. Relative protein levels were calculated. (n=3)

Supplemental figure 3. Flow cytometry gating strategy for Figure.4 and Figure.6E.

Supplemental figure 4. Original Western blots for Figure.5 A.B.C and Supplemental figure 2.

## Data Availability

The datasets generated and/or analyzed during the current study are available from the corresponding authors on reasonable request.

## References

[1] Lycett SJ, Duchatel F, Digard P. A brief history of bird flu. Philos Trans R Soc Lond B Biol Sci. 2019;374:20180257. 31056053 10.1098/rstb.2018.0257 PMC6553608

[2] W. H. Organization: Influenza is on the rise; how do I prevent it? 2021. https://www.who.int/news/item/01-10-2021-influenza-is-on-the-rise-how-do-i-prevent-it

[3] Liu YN, Zhang YF, Xu Q, Qiu Y, Lu QB, Wang T, et al. Infection and co-infection patterns of community-acquired pneumonia in patients of different ages in China from 2009 to 2020: a national surveillance study. Lancet Microbe. 2023;4:e330–e339. 37001538 10.1016/S2666-5247(23)00031-9 PMC12514336

[4] Blyth CC, Webb SAR, Kok J, Dwyer DE, van Hal SJ, Foo H, et al. The impact of bacterial and viral co-infection in severe influenza. Influenza Other Respir Viruses. 2013;7:168–76. 22487223 10.1111/j.1750-2659.2012.00360.x PMC5006004

[5] Britto C, Mohorianu I, Yeung T, Cheung E, Novak T, Hall MW, et al. Host respiratory transcriptome signature associated with poor outcome in children with influenza-*Staphylococcus aureus* Pneumonia. J Infect Dis. 2022;226:1286–94. 35899844 10.1093/infdis/jiac325 PMC10233493

[6] Teng F, Liu X, Guo S-B, Li Z, Ji W-Q, Zhang F, et al. Community-acquired bacterial co-infection predicts severity and mortality in influenza-associated pneumonia admitted patients. J Infect Chemother. 2019;25:129–36. 30448361 10.1016/j.jiac.2018.10.014

[7] Wang S, Yang J, Sun W, Tao Y. Severe necrotizing tracheobronchitis caused by influenza B and methicillin-resistant *Staphylococcus aureus* co-infection in an immunocompetent patient. Ann Clin Microbiol Antimicrob. 2024;23:55. 38886754 10.1186/s12941-024-00715-1 PMC11184759

[8] Chung DR, Huh K. Novel pandemic influenza A (H1N1) and community-associated methicillin-resistant *Staphylococcus aureus* pneumonia. Expert Rev Anti-infective Ther. 2015;13:197–207. 10.1586/14787210.2015.999668 25578884

[9] Rice TW, Rubinson L, Uyeki TM, Vaughn FL, John BB, Miller RR, et al. Critical illness from 2009 pandemic influenza A virus and bacterial coinfection in the United States. Crit Care Med. 2012;40:1487–98. 22511131 10.1097/CCM.0b013e3182416f23 PMC3653183

[10] Oliva J and Terrier, O. Viral and bacterial co-infections in the lungs: dangerous liaisons. Viruses. 2021;13:1725. 10.3390/v13091725 PMC8472850 34578306

[11] Pittet LA, Hall-Stoodley L, Rutkowski MR, Harmsen AG. Influenza virus infection decreases tracheal mucociliary velocity and clearance of Streptococcus pneumoniae. Am J Respir Cell Mol Biol. 2010;42:450–60. 19520922 10.1165/rcmb.2007-0417OC PMC2848738

[12] Snelgrove RJ, Godlee A, Hussell T. Airway immune homeostasis and implications for influenza-induced inflammation. Trends Immunol. 2011;32:328–34. 21612981 10.1016/j.it.2011.04.006

[13] Pociask DA, Scheller EV, Mandalapu S, McHugh KJ, Enelow RI, Fattman CL, et al. IL-22 is essential for lung epithelial repair following influenza infection. Am J Pathol. 2013;182:1286–96. 23490254 10.1016/j.ajpath.2012.12.007 PMC3620404

[14] AS Neupane, M Willson, AK Chojnacki, F Vargas E Silva Castanheira, C Morehouse, A Carestia, et al. Patrolling alveolar macrophages conceal bacteria from the immune system to maintain homeostasis. Cell 2020;183:110–125. 10.1016/j.cell.2020.08.020 32888431

[15] M Peng, C Zhao, F Lu, X Zhang, X Wang, L He, et al. Role of Nedd4L in macrophage pro-inflammatory polarization induced by influenza A virus and lipopolysaccharide stimulation. Microorganisms. 2024;12:1291. 10.3390/microorganisms12071291 PMC11279021 39065060

[16] Shen X, Sun C, Cheng Y, Ma D, Sun Y, Lin Y, et al. cGAS Mediates Inflammation by Polarizing Macrophages to M1 Phenotype via the mTORC1 Pathway. J Immunol. 2023;210:1098–107. 36881861 10.4049/jimmunol.2200351

[17] Xu Y, Zhang C, Cai D, Zhu R, Cao Y. Exosomal miR-155-5p drives widespread macrophage M1 polarization in hypervirulent Klebsiella pneumoniae-induced acute lung injury via the MSK1/p38-MAPK axis. Cell Mol Biol Lett. 2023;28:92. 37953267 10.1186/s11658-023-00505-1 PMC10641976

[18] Hang DTT, Choi E-J, Song J-Y, Kim S-E, Kwak J, Shin Y-K. Differential effect of prior influenza infection on alveolar macrophage phagocytosis of *Staphylococcus aureus* and Escherichia coli: involvement of interferon-gamma production. Microbiol Immunol. 2011;55:751–9. 21895747 10.1111/j.1348-0421.2011.00383.x

[19] Verma AK, Bansal S, Bauer C, Muralidharan A, Sun K. Influenza infection induces alveolar macrophage dysfunction and thereby enables noninvasive streptococcus pneumoniae to cause deadly pneumonia. J Immunol. 2020;205:1601–7. 32796026 10.4049/jimmunol.2000094 PMC7484308

[20] Taban Q, Mumtaz PT, Masoodi KZ, Haq E, Ahmad SM. Scavenger receptors in host defense: from functional aspects to mode of action. Cell Commun Signal. 2022;20:2. 34980167 10.1186/s12964-021-00812-0 PMC8721182

[21] Fan EKY, Fan J. Regulation of alveolar macrophage death in acute lung inflammation. Respir Res. 2018;19:50. 29587748 10.1186/s12931-018-0756-5 PMC5872399

[22] Yuan J, Ofengeim D. A guide to cell death pathways. Nat Rev Mol Cell Biol. 2024;25:379–95. 38110635 10.1038/s41580-023-00689-6

[23] Kuang L, Wu Y, Shu J, Yang J, Zhou H, Huang X. Pyroptotic macrophage-derived microvesicles accelerate formation of neutrophil extracellular traps via GSDMD-N-expressing mitochondrial transfer during sepsis. Int J Biol Sci. 2024;20:733–50. 38169726 10.7150/ijbs.87646 PMC10758106

[24] He X, Qian Y, Li Z, Fan EK, Li Y, Wu L, et al. TLR4-upregulated IL-1β and IL-1RI promote alveolar macrophage pyroptosis and lung inflammation through an autocrine mechanism. Sci Rep. 2016;6:31663. 27526865 10.1038/srep31663 PMC4985817

[25] H Kawasuji, K Nagaoka, Y Tsuji, K Kimoto, Y Takegoshi, M Kaneda, et al. Effectiveness and safety of linezolid versus vancomycin, teicoplanin, or daptomycin against methicillin-resistant *Staphylococcus aureus* bacteremia: a systematic review and meta-analysis. Antibiotics 2023;12:697. 10.3390/antibiotics12040697 PMC10135165 37107059

[26] Boianelli A, Sharma-Chawla N, Bruder D, Hernandez-Vargas EA. Oseltamivir PK/PD modeling and simulation to evaluate treatment strategies against influenza-pneumococcus coinfection. Front Cell Infect Microbiol. 2016;6:60. 27379214 10.3389/fcimb.2016.00060 PMC4906052

[27] C Malainou, SM Abdin, N Lachmann, U Matt and S Herold. Alveolar macrophages in tissue homeostasis, inflammation, and infection: evolving concepts of therapeutic targeting. J Clin Investig. 2023;133:e170501. 10.1172/JCI170501 PMC10541196 37781922

[28] Hu JJ, Liu X, Xia S, Zhang Z, Zhang Y, Zhao J, et al. FDA-approved disulfiram inhibits pyroptosis by blocking gasdermin D pore formation. Nat Immunol. 2020;21:736–45. 32367036 10.1038/s41590-020-0669-6 PMC7316630

[29] Wei Y, You Y, Zhang J, Ban J, Min H, Li C, et al. Crystalline silica-induced macrophage pyroptosis interacting with mitophagy contributes to pulmonary fibrosis via modulating mitochondria homeostasis. J Hazard Mater. 2023;454:131562. 37148789 10.1016/j.jhazmat.2023.131562

[30] Silva CMS, Wanderley CWS, Veras FP, Sonego F, Nascimento DC, Gonçalves AV, et al. Gasdermin D inhibition prevents multiple organ dysfunction during sepsis by blocking NET formation. Blood. 2021;138:2702–13. 34407544 10.1182/blood.2021011525 PMC8703366

[31] JC Kash, K-A Walters, AS Davis, A Sandouk, LM Schwartzman, BW Jagger, et al. Lethal synergism of 2009 pandemic H1N1 influenza virus and Streptococcus pneumoniae coinfection is associated with loss of murine lung repair responses. MBio. 2011;2:e00172-11. 10.1128/mBio.00172-11 PMC3175626 21933918

[32] Kash JC, Taubenberger JK. The role of viral, host, and secondary bacterial factors in influenza pathogenesis. Am J Pathol. 2015;185:1528–36. 25747532 10.1016/j.ajpath.2014.08.030 PMC4450310

[33] Montgomery MT, Ortigoza M, Loomis C, Weiser JN. Neuraminidase-mediated enhancement of Streptococcus pneumoniae colonization is associated with altered mucus characteristics and distribution. MBio. 2025;16:e0257924. 39660923 10.1128/mbio.02579-24 PMC11708046

[34] A Biram, J Liu, H Hezroni, N Davidzohn, D Schmiedel, E Khatib-Massalha, et al. Bacterial infection disrupts established germinal center reactions through monocyte recruitment and impaired metabolic adaptation. Immunity 2022;55:442–58. 10.1016/j.immuni.2022.01.013 35182483

[35] Spottiswoode N, Tsitsiklis A, Chu VT, Phan HV, DeVoe C, Love C, et al. Microbial dynamics and pulmonary immune responses in COVID-19 secondary bacterial pneumonia. Nat Commun. 2024;15:9339. 39472555 10.1038/s41467-024-53566-x PMC11522429

[36] Xu M-M, Kang J-Y, Wang Q-Y, Zuo X, Tan Y-Y, Wei Y-Y, et al. Melatonin improves influenza virus infection-induced acute exacerbation of COPD by suppressing macrophage M1 polarization and apoptosis. Respir Res. 2024;25:186. 38678295 10.1186/s12931-024-02815-0 PMC11056066

[37] Zhang Y, Li J, Qiu Z, Huang L, Yang S, Li J, et al. Insights into the mechanism of action of pterostilbene against influenza A virus-induced acute lung injury. Phytomed Int J Phytother Phytopharmacol. 2024;129:155534. 10.1016/j.phymed.2024.155534 38583346

[38] Yang Z, Wang J, Pi J, Hu D, Xu J, Zhao Y, et al. Identification and validation of genes related to macrophage polarization and cell death modes under mycobacterium tuberculosis infection. J Inflamm Res. 2024;17:1397–411. 38476473 10.2147/JIR.S448372 PMC10927374

[39] Jiao Y, Zhang T, Zhang C, Ji H, Tong X, Xia R, et al. Exosomal miR-30d-5p of neutrophils induces M1 macrophage polarization and primes macrophage pyroptosis in sepsis-related acute lung injury. Crit Care. 2021;25:356. 34641966 10.1186/s13054-021-03775-3 PMC8507252

[40] X Chen, R Wu, L Li, Y Zeng, J Chen, M Wei, et al. Pregnancy-induced changes to the gut microbiota drive macrophage pyroptosis and exacerbate septic inflammation. Immunity. 2023;56:336–52. 10.1016/j.immuni.2023.01.015 36792573

[41] Xu N, Jiang J, Jiang F, Dong G, Meng L, Wang M, et al. CircCDC42-encoded CDC42-165aa regulates macrophage pyroptosis in Klebsiella pneumoniae infection through Pyrin inflammasome activation. Nat Commun. 2024;15:5730. 38977695 10.1038/s41467-024-50154-x PMC11231140

[42] Wei Y, Lan B, Zheng T, Yang L, Zhang X, Cheng L, et al. GSDME-mediated pyroptosis promotes the progression and associated inflammation of atherosclerosis. Nat Commun. 2023;14:929. 36807553 10.1038/s41467-023-36614-w PMC9938904

[43] Qin X, Zhou Y, Jia C, Chao Z, Qin H, Liang J, et al. Caspase-1-mediated extracellular vesicles derived from pyroptotic alveolar macrophages promote inflammation in acute lung injury. Int J Biol Sci. 2022;18:1521–38. 35280692 10.7150/ijbs.66477 PMC8898368

[44] J Lin, F Li, J Jiao, Y Qian, M Xu, F Wang, et al. Quercetin, a natural flavonoid, protects against hepatic ischemia-reperfusion injury via inhibiting Caspase-8/ASC dependent macrophage pyroptosis. J Adv Res 2024;70:555–69. 10.1016/j.jare.2024.05.010 PMC11976413 38735388

[45] Zhou X, Qin M, He L, Zhang Y, Liu A, Chen D, et al. Geraniin restricts inflammasome activation and macrophage pyroptosis by preventing the interaction between ASC and NLRP3 to exert anti-inflammatory effects. Int Immunopharmacol. 2024;129:111656. 38340422 10.1016/j.intimp.2024.111656

[46] Shi P, Du Y, Zhang Y, Yang B, Guan Q, Jing Y, et al. Ubiquitin-independent degradation of Bim blocks macrophage pyroptosis in sepsis-related tissue injury. Cell Death Dis. 2024;15:703. 39349939 10.1038/s41419-024-07072-z PMC11442472

[47] Liang W, Wei R, Zhu X, Li J, Lin A, Chen J, et al. Downregulation of HMGB1 carried by macrophage-derived extracellular vesicles delays atherosclerotic plaque formation through Caspase-11-dependent macrophage pyroptosis. Mol Med. 2024;30:38. 38493291 10.1186/s10020-023-00753-z PMC10943908

[48] Tao Y, Xu X, Yang B, Zhao H, Li Y. Mitigation of sepsis-induced acute lung injury by BMSC-derived exosomal miR-125b-5p through STAT3-mediated suppression of macrophage pyroptosis. Int J Nanomed. 2023;18:7095–113. 10.2147/IJN.S441133 PMC10693758 38050472

[49] Huang D, Yao Y, Lou Y, Kou L, Yao Q, Chen R. Disulfiram and cancer immunotherapy: advanced nano-delivery systems and potential therapeutic strategies. Int J Pharmaceutics: X. 2024;8:100307. 10.1016/j.ijpx.2024.100307 PMC11638648 39678262

[50] Winchell CG, Mishra BB, Phuah JY, Saqib M, Nelson SJ, Maiello P, et al. Evaluation of IL-1 blockade as an adjunct to linezolid therapy for tuberculosis in Mice and Macaques. Front Immunol. 2020;11:891. 32477361 10.3389/fimmu.2020.00891 PMC7235418

[51] X Li, W Ding, Y Lu, H Zhu, W Bao, Y Liu, et al. An anti-complement homogeneous polysaccharide from Houttuynia cordata ameliorates acute pneumonia with H1N1 and MRSA coinfection through rectifying Treg/Th17 imbalance in the gut–lung axis and NLRP3 inflammasome activation. Acta Pharmaceutica Sinica B. 2025;15:3073–91. 10.1016/j.apsb.2025.04.008 PMC12254813 40654358

