# Supplementary figures and images for: Excessive pyroptosis mediates the exacerbation of pneumonia caused by low-lethality influenza virus and secondary MRSA co-infection
Source: Cell Death Discov. 2026 Apr 2;12:216. doi: 10.1038/s41420-026-03031-z (PMC13172521; doi:10.1038/s41420-026-03031-z)

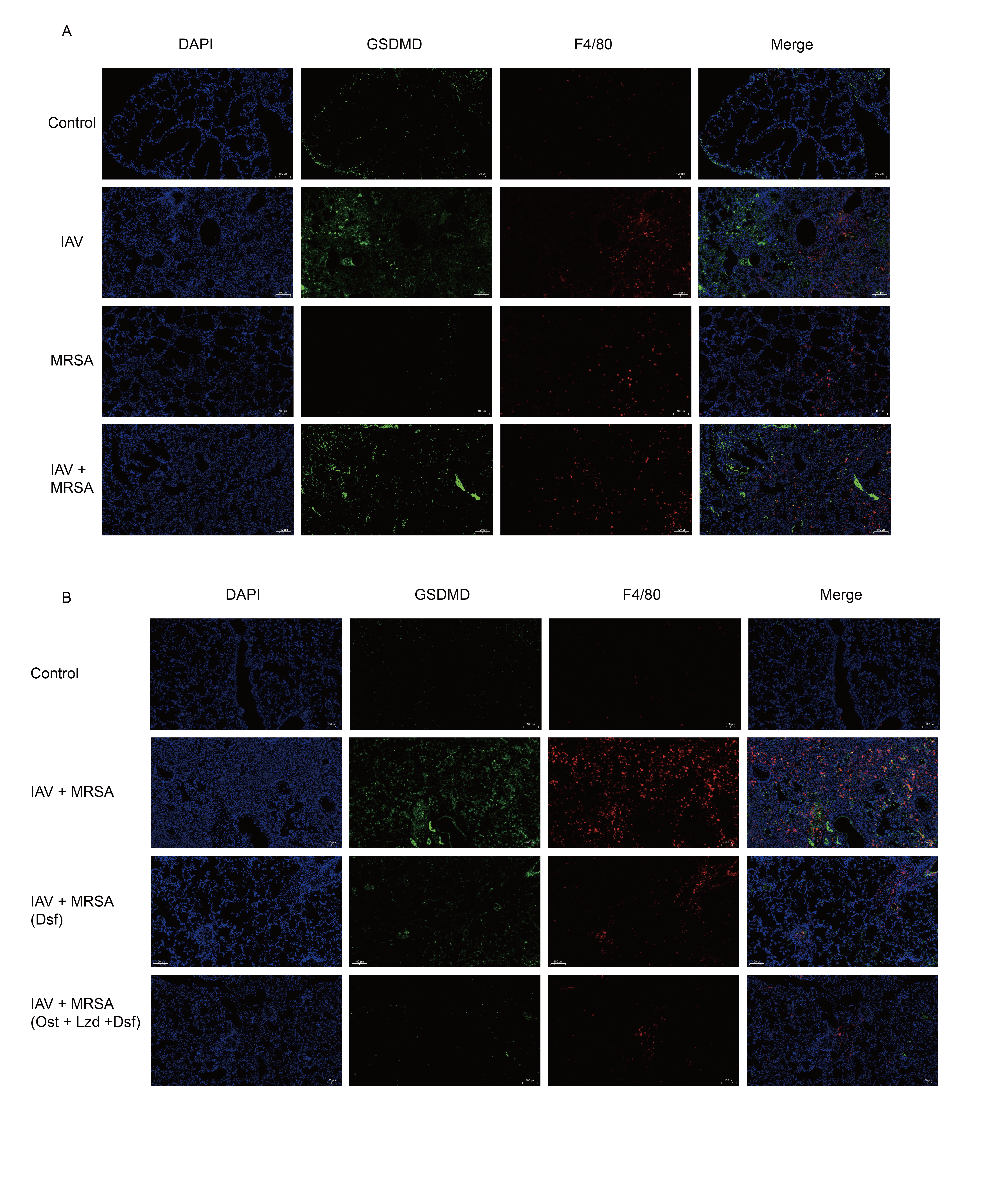

Supplement: Supplementary file 1 — Supplemental figure 1. Immunofluorescence image for Figure.5D and Figure.7F under 20× magnification. [file 41420_2026_3031_MOESM1_ESM.png]

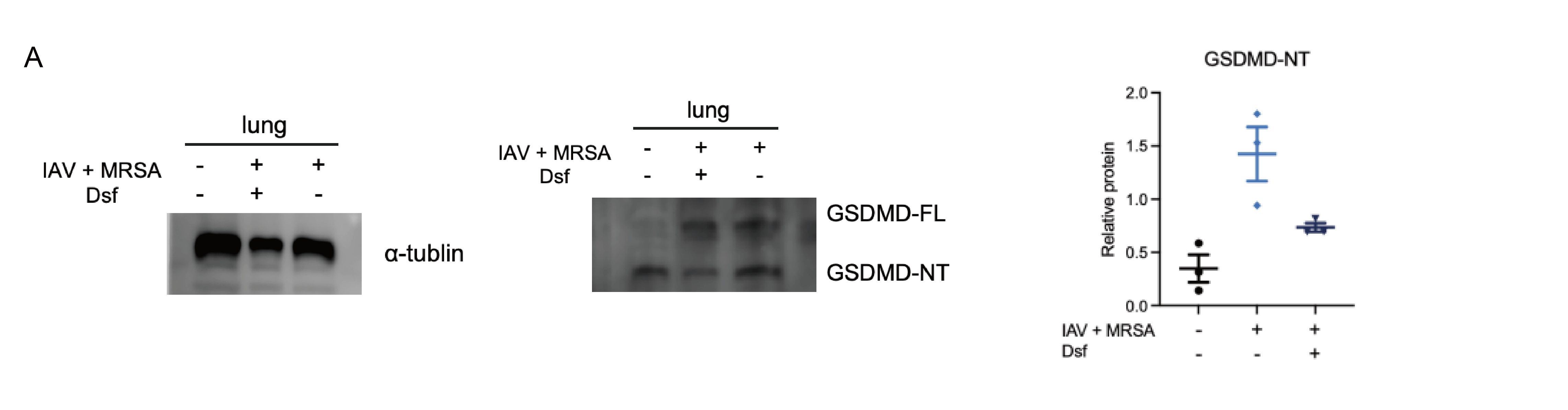

Supplement: Supplementary file 2 — Supplemental figure 2. Lung lysates were immunoblotted with anti-GSDMD and anti-α-tubulin antibodies. Relative protein levels were calculated. (n=3) [file 41420_2026_3031_MOESM2_ESM.png]

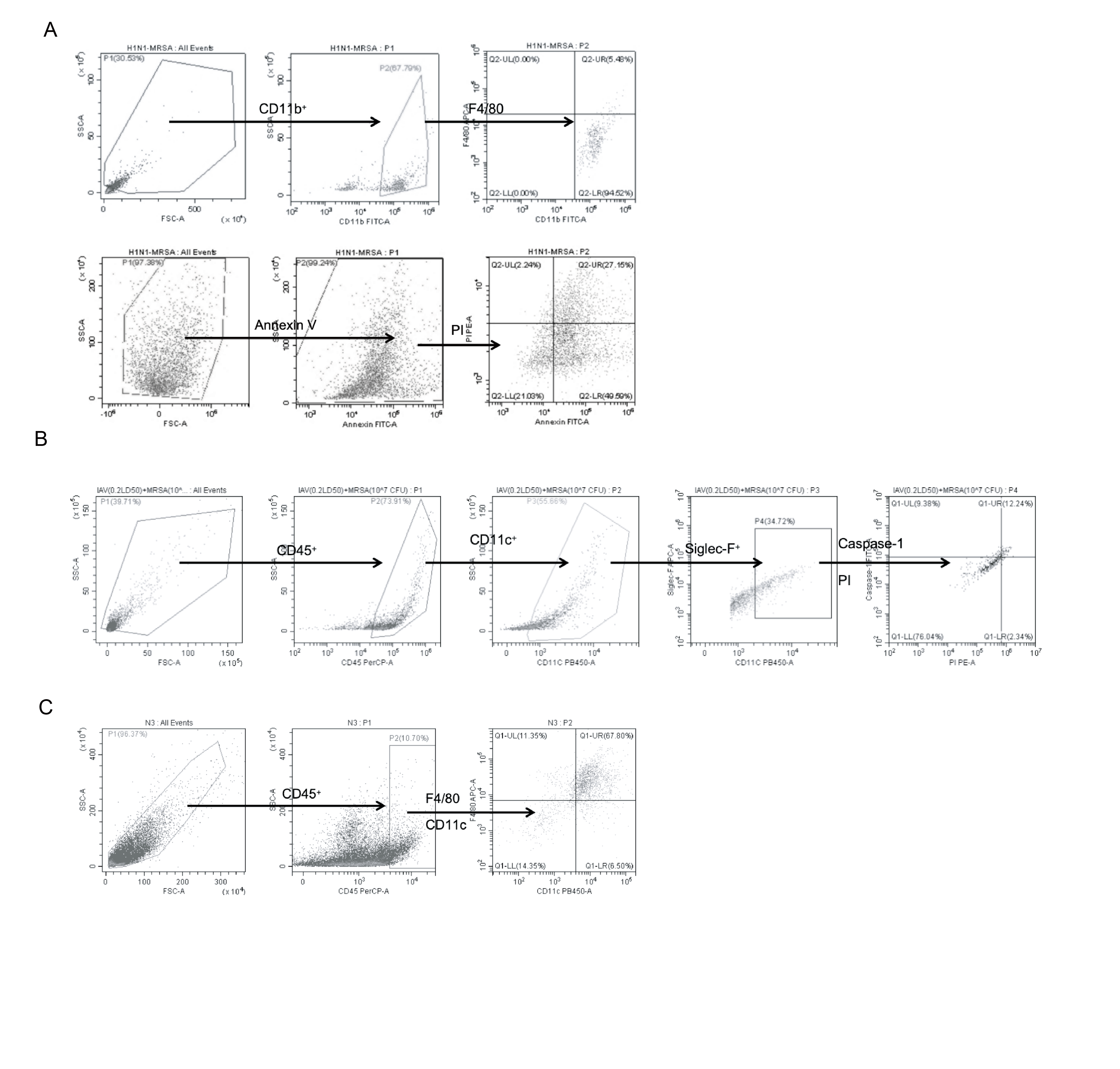

Supplement: Supplementary file 3 — Supplemental figure 3. Flow cytometry gating strategy for Figure.4 and Figure.6E. [file 41420_2026_3031_MOESM3_ESM.png]

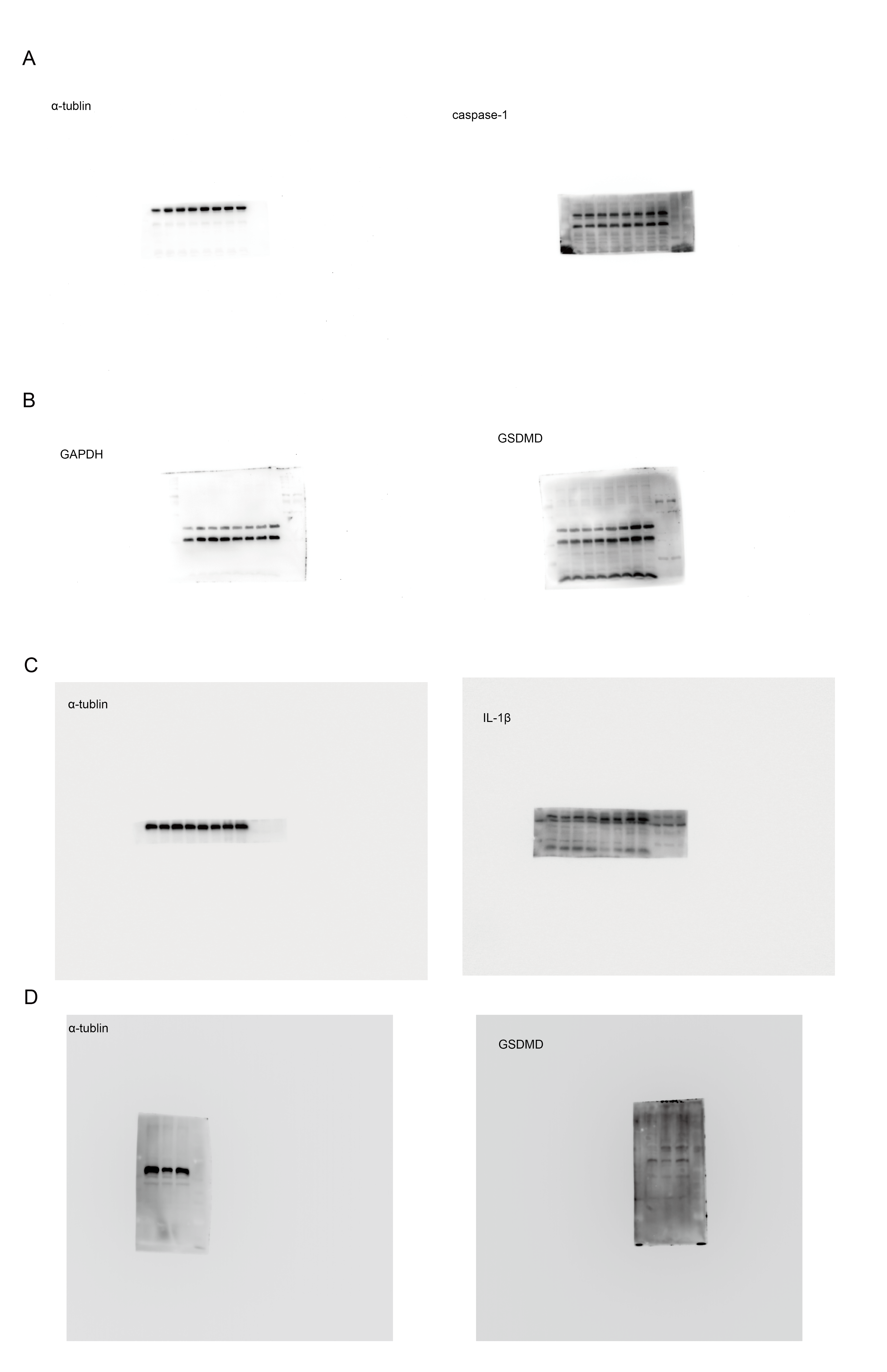

Supplement: Supplementary file 4 — Supplemental figure 4. Original Western blots for Figure.5 A.B.C and Supplemental figure 2. [file 41420_2026_3031_MOESM4_ESM.png]
